# Predictors of post‐operative decline in knee flexion after cruciate‐retaining total knee arthroplasty

**DOI:** 10.1002/jeo2.70419

**Published:** 2025-09-09

**Authors:** Tatsuya Kubo, Tsuneari Takahashi, Katsushi Takeshita

**Affiliations:** ^1^ Department of Orthopaedic Surgery Shin‐Oyama City Hospital Oyama Japan; ^2^ Department of Orthopaedics Jichi Medical University Shimotsuke Japan

**Keywords:** flexion range of motion, knee osteoarthritis, predictor, receiver operating characteristic curve

## Abstract

**Purpose:**

This study aimed to identify risk factors associated with deterioration in knee flexion range of motion (ROM) following cruciate‐retaining total knee arthroplasty (CR‐TKA).

**Methods:**

A total of 129 consecutive patients who underwent CR‐TKA and completed a 2‐year follow‐up were included. A decrease of ≥9° in flexion ROM was considered clinically significant. Patients were classified into two groups based on the change in flexion ROM at 2 years post‐operatively: Group D (≥9° decrease; *n* = 44) and Group C (<9° decrease; *n* = 85). Demographic data were compared using Student's *t* test or Fisher's exact test. Logistic regression analysis was performed to identify factors associated with ROM deterioration. Receiver operating characteristic (ROC) curve analysis was conducted to determine cut‐off values for the identified factors.

**Results:**

Demographic characteristics were comparable between the two groups. Preoperative flexion ROM was significantly higher in Group D than in Group C (128.6 ± 9.5° vs. 115.5 ± 12.3°, *p* < 0.001). Logistic regression analysis revealed that greater preoperative flexion ROM (odds ratio [OR], 1.18; 95% confidence interval [CI], 1.10–1.27; *p* < 0.001), a larger distal medial femoral resection (DMFR) (OR, 1.98; 95% CI, 1.14–3.43; *p* = 0.015) and lower anterior‐posterior translation at 30° of flexion 1 year post‐operatively (1y30AP) (OR, 0.73; 95% CI, 0.57–0.93; *p* = 0.012) were independently associated with a ≥9° reduction in flexion ROM. ROC analysis identified cut‐off values of >130.0° for preoperative flexion ROM (AUC: 0.80, 95% CI: 0.72–0.88), >7.5 mm for DMFR (AUC: 0.60, 95% CI: 0.50–0.71) and <5.1 mm for 1y30AP (AUC: 0.62, 95% CI: 0.51–0.72).

**Conclusions:**

Preoperative flexion ROM, DMFR and 1y30AP were associated with worse flexion ROM at 2 years following CR‐TKA.

**Level of Evidence:**

Level III, retrospective comparative study.

**Abbreviations**
1y30AP: anterior‐posterior translation at 30° flexion 1 year post‐operatively
ACL: anterior cruciate ligament
AP: anterior‐posterior
AUC: area under the curve
BMI: body mass index
CI: confidence interval
CR‐TKA: cruciate‐retaining total knee arthroplasty
DMFR: distal medial femoral resection
HKA: hip–knee–ankle angle
MCID: minimal clinically important difference
NPV: negative predictive value
OR: odds ratio
PCL: posterior cruciate ligament
PPV: positive predictive value
PROMs: patient‐reported outcome measures
ROC: receiver operating characteristic
ROM: range of motion
SD: standard deviation
TKA: total knee arthroplasty

## INTRODUCTION

Total knee arthroplasty (TKA) is a well‐established surgical option for treating end‐stage knee osteoarthritis [21]. While the primary goal of TKA is pain relief, restoration of knee function is also critical for achieving favourable clinical outcomes and overall success [20]. Despite advances in implant design, surgical technique and perioperative management, approximately 20% of patients remain dissatisfied following TKA, a figure that has remained relatively unchanged over time [2, 8].

Range of motion (ROM) is closely associated with functional outcomes and patient satisfaction after TKA [5, 10]. Specific degrees of knee flexion are necessary for various daily activities: 67° for walking, 83° for descending stairs, 90° for ascending stairs, 105° for rising from a chair and 115° for standing up from a sofa [16]. Limitations in post‐operative flexion may hinder these activities and contribute to patient dissatisfaction, particularly when patients experience decreased activity levels and are unable to resume previously anticipated functions [18, 20]. Achieving an adequate ROM and avoiding post‐operative deterioration of flexion are therefore essential goals of TKA [10]. The primary aim of this study was to identify risk factors for worsening flexion ROM following TKA. The secondary aim was to determine cut‐off values for these risk factors.

## METHODS

### Study design and patients

This retrospective study of 129 consecutive patients who underwent cruciate‐retaining TKA (CR‐TKA) using the ATTUNE system (DePuy Synthes) between July 2018 and November 2022, with a minimum follow‐up period of 2 years. All procedures were performed by a single experienced arthroplasty surgeon using a mechanically aligned technique, in accordance with the manufacturer's guidelines. Bone cuts were made using a measured resection technique, and soft tissue balance was achieved through selective release of the surrounding tissues. The indication for TKA was based on persistent pain and functional decline despite adequate conservative treatment, corroborated by radiographic evidence of Kellgren–Lawrence Grade 3 or 4 osteoarthritis [15]. Patients with a history of TKA, anterior cruciate ligament (ACL) reconstruction, posterior cruciate ligament (PCL) reconstruction or knee osteotomy, which could potentially affect post‐operative ROM, were excluded from the study. Based on previous studies, a change of 9° in knee flexion was defined as the minimal clinically important difference (MCID) [9, 26]. Accordingly, patients were divided into two groups: Group D included 44 patients who experienced a deterioration of ≥9° in flexion ROM at 2 years post‐operatively compared to baseline, while Group C (control group) comprised 85 patients without such a reduction.

### Evaluation items

The following patient data were collected: age, sex, hip–knee–ankle (HKA) angle (with positive values indicating varus alignment) and ROM in extension and flexion both preoperatively and post‐operatively (with negative values indicating extension limitation). ROM was measured in degrees (°) using a standard bilateral arm goniometer.

At 1 year post‐operatively, anterior‐posterior (AP) laxity of the knee was assessed using a Kneelax 3 arthrometer. Following the method described by Shi et al., anterior and posterior forces of 132 N were applied to the tibia at 30° and 90° of knee flexion, and the resulting AP laxity was recorded [25].

Intraoperatively, calipers were used to measure the medial and lateral resection amounts of the distal and dorsal femur and proximal tibia [29]. Macroscopic findings of the ACL were also documented and categorized as intact, injured with reduced synovial coverage and ligament laxity, or degenerated [28].

### Surgical procedure

All TKAs were performed using a midvastus approach with the use of an air tourniquet. The ACL was resected in all cases, while the PCL was fully preserved. Patellar resurfacing was not performed in any case. Mechanical alignment was achieved by cutting the femur and tibia to create a rectangular flexion–extension gap perpendicular to the mechanical axis, resulting in neutral coronal alignment. The distal femur was cut using an intramedullary alignment system. A cutting guide was employed for the posterior femoral cut to align the osteotomy line parallel to the surgical epicondylar axis and perpendicular to Whiteside's line [30]. The proximal tibia was cut using an extramedullary alignment system. No collateral ligament release was performed, thereby maintaining the natural balance between flexion and extension. All final components were implanted using bone cement.

### Statistical analysis

Data are presented as mean (standard deviation [SD]) with a significance level of *p* < 0.05. All statistical analyses were performed using EZR software [13]. Between‐group comparisons of demographic variables were conducted using the Student's *t* test or Fisher's exact test, as appropriate. Logistic regression analysis using the forced entry method was performed to identify factors associated with a ≥9° reduction in flexion ROM. Receiver operating characteristic (ROC) curve analysis was used to determine optimal cut‐off values for these predictors, along with their positive and negative predictive values (PPV and NPV).

A post hoc power analysis was performed using G*Power software. Based on an alpha level of 0.05, power of 0.80, and the sample sizes of both groups, the calculated effect size was 0.524 [6].

### Figure generation

**Figure 1:**
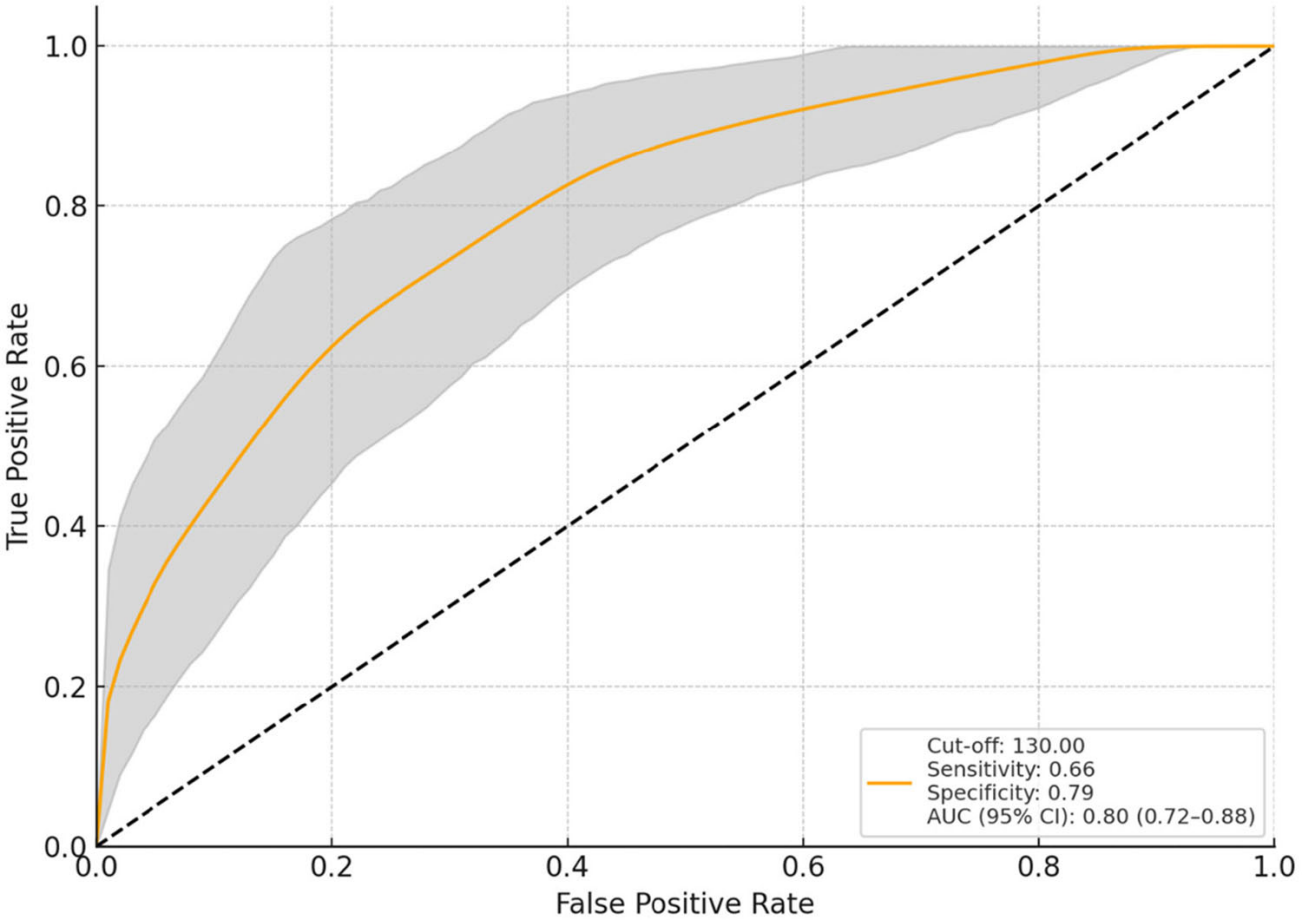
Predictive performance of preoperative flexion ROM for 2‐year flexion ROM deterioration: ROC curve with 95% confidence interval. AUC, area under the curve; ROC, receiver operating characteristic; ROM, range of motion.

**Figure 2:**
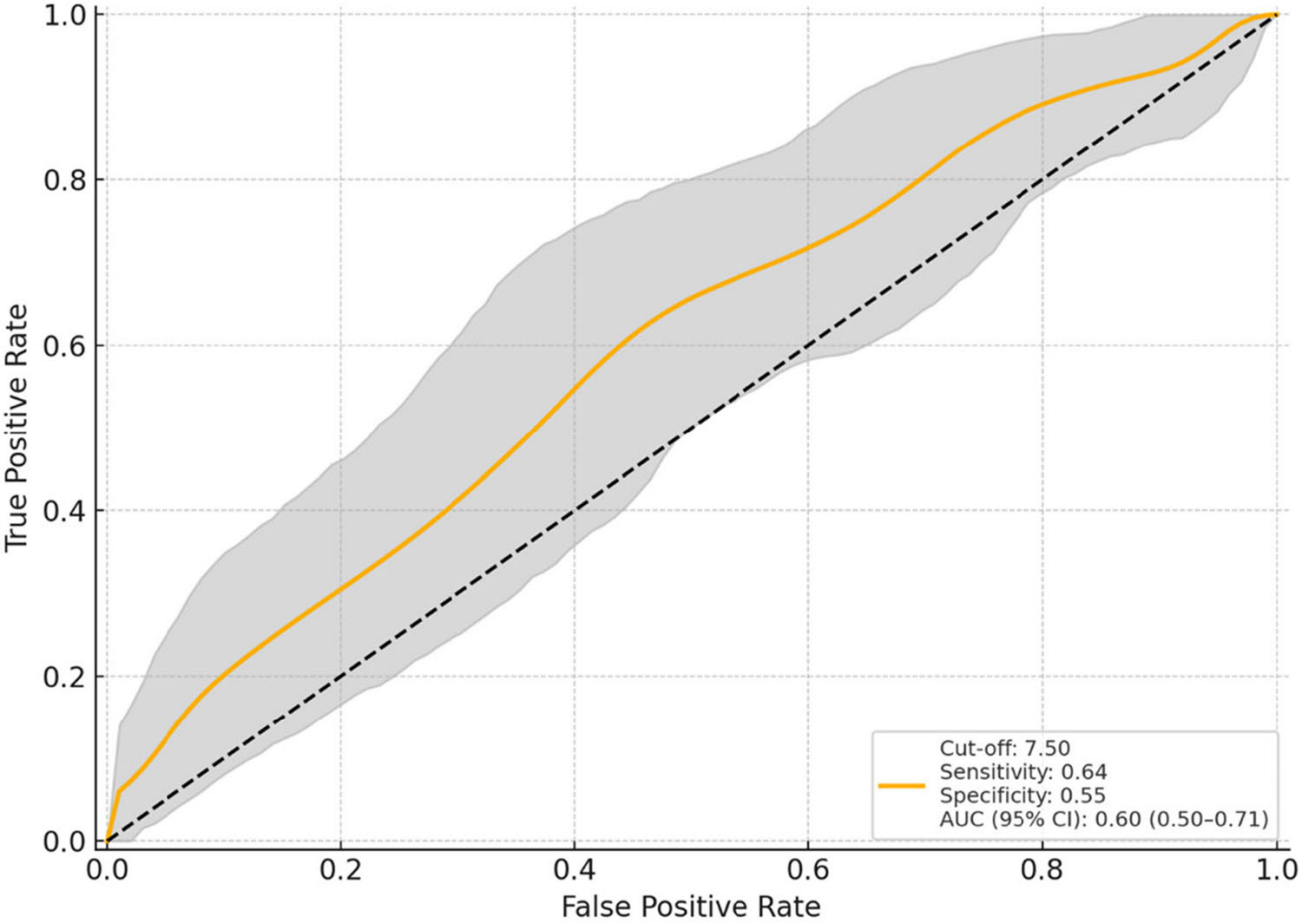
Predictive performance of medial distal femoral resection for 2‐year flexion ROM deterioration: ROC curve with 95% confidence interval. AUC, area under the curve; ROC, receiver operating characteristic; ROM, range of motion.

**Figure 3:**
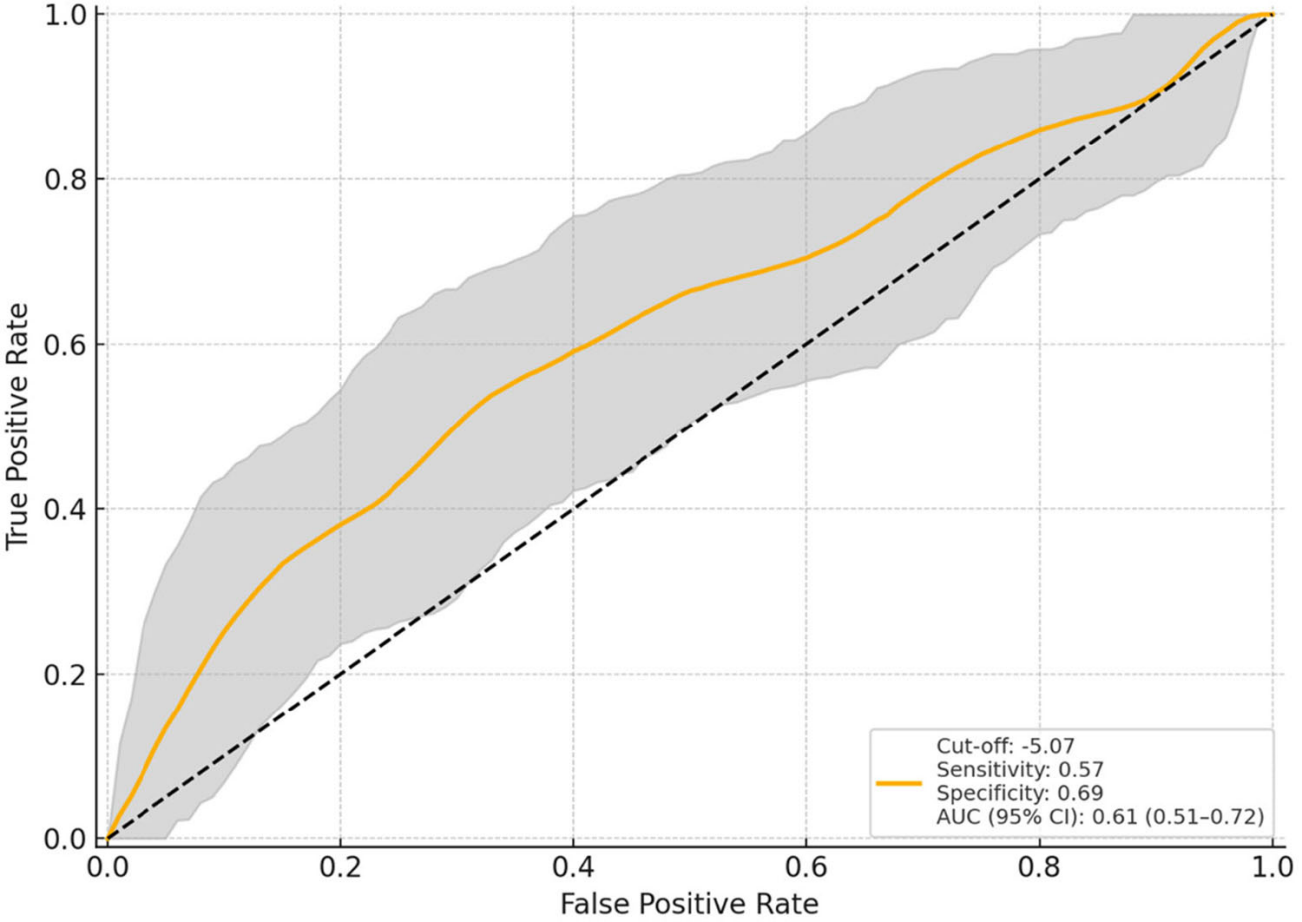
Predictive performance of anteroposterior translation at 30° flexion 1 year post‐operatively, for 2‐year flexion ROM deterioration: ROC curve with 95% confidence interval. 1y30°AP; anteroposterior translation at 30° flexion 1 year post‐operatively, AUC, area under the curve; ROC, receiver operating characteristic; ROM, range of motion.

Figures 1, 2, 3, which present the ROC curves, were generated using Python to supplement the statistical analyses performed with EZR. Because EZR does not provide 95% confidence intervals (CIs) for ROC curves, these intervals were calculated and plotted using Python.

## RESULTS

### Comparison of patients' characteristics

The average age of patients was 72.6 years (SD = 7.5). Of these, 76.7% were female. The average preoperative HKA and knee extension and flexion ROM were recorded as 12.4° (SD = 6.7°), −8.7° (SD = 7.0°) and 120.0° (SD = 13.0°), respectively (see Table 1). Two years post‐operatively, the average flexion measured 118.2° (SD = 10.9°), with a paired *t* test indicating no significant difference between preoperative and 2‐year post‐operative flexion (*p* = 0.145). A weak correlation was identified between preoperative and 2‐year post‐operative flexion (*r* = 0.34, 95% CI = 0.18–0.49, *p* < 0.01).

**Table 1:** Patients' demographics.

Variable	Value
Number of patients	129
Age (years)	72.6 (7.5)
Female (%)	99 (76.7)
Height (cm)	155.9 (7.8)
Weight (kg)	62.2 (11.7)
BMI (kg/m^2^)	25.8 (3.9)
Preoperative HKA (°)	12.4 (6.7)
Extension	
Preoperative (°)	−8.7 (7.0)
Post‐operative (°)	−1.3 (3.2)
Flexion	
Preoperative (°)	120.0 (13.0)
Post‐operative (°)	118.2 (10.9)

*Note*: Values are expressed as the number of patients (%) or mean (SD).

Abbreviations: BMI, body mass index; HKA, hip–knee–ankle angle; SD, standard deviation.

There were no notable differences between Groups D and C regarding age, gender, preoperative ROM in extension, preoperative HKA, operative time and ACL conditions. However, significant variances were observed in preoperative flexion ROM (128.6° [SD = 9.5°] in Group D vs. 115.5° [SD = 12.3°] in Group C, *p* < 0.001), distal medial femoral resection (DMFR) amount (7.6 mm [SD = 1.3] in Group D vs. 7.1 [SD = 1.3] in Group C, *p* = 0.036), and the anteroposterior translation amount in 30° flexion 1 year post‐operatively (1y30AP) (5.4 [SD = 2.6] in Group D vs. 6.4 [SD = 2.6] in Group C, *p* = 0.035) after 1 year (Table 2).

**Table 2:** Between‐group comparison of patients with and without reduced knee flexion ROM.

Variable	Group D (*n* = 44)	Group C (*n* = 85)	*p* value
Age (years)	73.0 (7.1)	72.4 (7.6)	0.657
Female (%)	34 (77.3)	65 (76.5)	1
Height (cm)	153.6 (8.3)	155.6 (7.5)	0.173
Weight (kg)	59.8 (9.1)	63.4 (12.7)	0.094
BMI (kg/m^2^)	25.3 (3.1)	26.1 (4.3)	0.294
Preoperative HKA (°)	11.3 (6.7)	12.9 (6.7)	0.211
Extension			
Preoperative (°)	−7.3 (6.4)	−9.5 (7.2)	0.091
Post‐operative (°)	−0.9 (2.2)	−1.5 (3.5)	0.292
Flexion			
Preoperative (°)	128.6 (9.5)	115.5 (12.3)	<0.001
Post‐operative (°)	112.5 (11.1)	121.1 (9.6)	<0.001
Operative time (min)	96.1 (11.7)	97.0 (17.3)	0.763
ACL conditions (normal/diminished/vanished)	20/18/6	25/43/17	0.205
Bony resection amounts			
Distal medial femur (mm)	7.6 (1.3)	7.1 (1.3)	0.036
Distal lateral femur (mm)	7.1 (1.6)	6.8 (1.3)	0.319
Dorsal medial femur (mm)	9.3 (2.0)	9.5 (1.8)	0.428
Dorsal lateral femur (mm)	6.8 (1.7)	7.0 (1.4)	0.337
Proximal medial tibia (mm)	4.3 (2.4)	4.0 (2.3)	0.487
Proximal lateral tibia (mm)	10.5 (1.6)	11.0 (1.9)	0.155
AP translation 1 year after TKA			
In 30° flexed position (mm)	5.4 (2.6)	6.4 (2.6)	0.035
In 90° flexed position (mm)	2.5 (2.2)	3.0 (1.8)	0.246

*Note*: Values are expressed as the number of patients (%) or mean (SD).

Abbreviations: ACL, anterior cruciate ligament; AP, anterior‐posterior; BMI, body mass index; HKA, hip–knee–ankle angle; ROM, range of motion; SD, standard deviation; TKA, total knee arthroplasty.

### Multivariate analysis

In logistic regression analysis, the factors linked to a ≥9° decrease in ROM at 2 years post‐surgery compared to preoperative levels included preoperative flexion ROM (odds ratio [OR] = 1.18, 95% CI = 1.10–1.27, *p* < 0.001), the DMFR (OR = 1.98, 95% CI = 1.14–3.43, *p* = 0.015) and the 1y30AP (OR = 0.73, 95% CI = 0.57–0.93, *p* = 0.012) (Table 3). ROC curve analysis identified the cut‐off values for deteriorating ROM at 2 years post‐operatively: preoperative flexion ROM exceeding 130.0° (AUC = 0.80, 95% CI = 0.72–0.88), DMFR exceeding 7.5 mm (AUC = 0.60, 95% CI = 0.50–0.71) and 1y30AP falling below 5.1 mm (AUC = 0.62, 95% CI = 0.51–0.72) (Figures 1, 2, 3). For the cut‐off of preoperative flexion ROM > 130.0°, sensitivity was 0.659, specificity 0.788, PPV 0.617 and NPV 0.817. For DMFR > 7.5 mm, the sensitivity was 0.636, specificity 0.553, PPV 0.424 and NPV 0.746. For 1y30AP < 5.1 mm, the sensitivity was 0.568, specificity 0.690, PPV 0.490 and NPV 0.753.

**Table 3:** Multivariate logistic regression analysis of factors reducing knee flexion ROM at 2 years post‐operatively.

Variables	Odds ratio (95% CI)	*p* value
Age (years)	0.99 (0.92–1.07)	0.848
Gender	0.59 (0.14–2.56)	0.483
Height (cm)	8.58 (0.00–262.00)	0.366
Weight (kg)	0.95 (0.89–1.01)	0.126
Preoperative HKA (°)	1.13 (0.99–1.29)	0.061
Preoperative extension (°)	1.05 (0.96–1.16)	0.271
Preoperative flexion (°)	1.18 (1.10–1.27)	<0.001
Operative time (min)	1.01 (0.97–1.05)	0.537
ACL conditions	1.96 (0.63–6.12)	0.249
Bony resection amounts		
Distal medial femur (mm)	1.98 (1.14–3.43)	0.015
Distal lateral femur (mm)	1.14 (0.74–1.75)	0.544
Dorsal medial femur (mm)	1.20 (0.76–1.90)	0.423
Dorsal lateral femur (mm)	0.67 (0.38–1.18)	0.164
Proximal medial tibia (mm)	1.17 (0.89–1.53)	0.261
Proximal lateral tibia (mm)	1.17 (0.79–1.71)	0.434
AP translation 1 year after TKA		
In 30° flexed position (mm)	0.73 (0.57–0.93)	0.012
In 90° flexed position (mm)	0.85 (0.65–1.12)	0.241

Abbreviations: ACL, anterior cruciate ligament; AP, anterior‐posterior; BMI, body mass index; CI, confidence interval; HKA, hip–knee–ankle angle; ROM, range of motion; SD, standard deviation; TKA, total knee arthroplasty.

## DISCUSSION

This study aimed to identify risk factors associated with a decline in flexion ROM following CR‐TKA. The main findings demonstrated that preoperative knee flexion ROM, intraoperative DMFR and 1y30AP were associated with a post‐operative reduction in ROM exceeding 9° from baseline at 2 years. However, the area under the curve (AUC) values for DMFR (0.60) and 1y30AP (0.62) were relatively low. Sensitivity, specificity, PPV and NPV analyses also indicated limited predictive accuracy. Therefore, the clinical utility of DMFR and 1y30AP as reliable predictors for post‐operative flexion ROM decline remains inconclusive.

Regarding the effect of preoperative flexion ROM on post‐operative ROM, our results showed that if the preoperative flexion ROM was greater than 130°, the risk of flexion ROM at 2 years post‐operatively was at least 9° worse than the preoperative flexion ROM. Previous studies have reported a positive correlation between preoperative and post‐operative flexion ROM, with the correlation being particularly strong the poorer the preoperative flexion ROM [1, 22, 27]. It has also been reported that pre‐ and post‐operative change in flexion ROM is negatively correlated with preoperative flexion ROM [3]. These reports suggest that poor preoperative flexion ROM is more likely to improve post‐operatively and that a well‐flexed knee at baseline may have a reduced flexion ROM post‐operatively, consistent with our study results. From a clinical perspective, when the preoperative flexion ROM exceeds 130°, consideration may be given to using implant designs other than the CR type, in order to better preserve post‐operative flexion capacity.

Elevation of the joint line due to excessive distal femoral resection has been reported to reduce ROM, increase instability and cause anterior knee pain [7, 17, 23]. An elevated joint line can result in patellar baja, which in turn is associated with reduced ROM [4, 14]. A greater amount of DMFR was statistically associated with decreased post‐operative ROM in our study, possibly due to elevation of the joint surface. However, this association demonstrated limited predictive accuracy and should therefore be interpreted with caution.

The clinical relevance of AP stability during mid‐flexion remains uncertain. Matsumoto et al. reported an average APlaxity of 4.5 mm at 30° flexion following TKA, with similar findings noted in other studies [11, 19, 24]. Warren et al. demonstrated that anterior laxity below 5 mm was associated with greater extension lag and reduced knee ROM [31], while Ishii et al. reported optimal post‐operative outcomes with laxity under 6 mm [12]. In line with these reports, our findings showed that AP translation below 5.1 mm was statistically associated with reduced post‐operative ROM. However, the low predictive accuracy limits its clinical applicability.

### Limitations

This study has several limitations that should be considered when interpreting the findings. First, the AUC values for DMFR and 1y30AP as indicators of deterioration were relatively low (0.60 and 0.62, respectively), suggesting limited predictive accuracy. Second, all participants were Japanese and recruited from a single institution, which may limit the generalizability of the results to other populations and settings. Third, this was a retrospective study design, which inherently carries potential biases despite careful patient selection and surgical performance by a single experienced surgeon. Fourth, intraoperative caliper measurements were not formally validated for intra‐ or inter‐observer reliability, although measurements were consistently performed by the same surgeon using a caliper with 0.5‐mm resolution.

Additionally, the AP laxity test was conducted while participants were awake, possibly including reflexive muscle contractions that might affect measurement accuracy. Moreover, while this study focused on ROM as an outcome, patient‐reported outcome measures (PROMs) such as the Knee Injury and Osteoarthritis Outcome Score, Knee Society Score or Forgotten Joint Score were not assessed. Since ROM alone does not fully capture patient satisfaction or functional outcome, the absence of PROMs is a notable limitation that may affect the clinical interpretability of our findings.

Furthermore, no consensus exists on the clinically meaningful change in knee flexion angle after TKA. This study defined a change of 9° or more as clinically significant based on minimal clinically important changes reported for non‐surgical interventions for knee osteoarthritis and MCID after stroke. However, these thresholds were derived from different populations, necessitating further research to establish clinically meaningful criteria specific to TKA patients.

Despite these limitations and the potentially limited clinical significance, identifying factors associated with post‐operative decline in flexion ROM may provide valuable insights for surgeons and rehabilitation specialists, aiding clinical decision‐making regarding implant selection and management strategies.

## CONCLUSION

Preoperative flexion, greater DMFR and reduced anteroposterior laxity at 30° flexion were associated with a post‐operative decline in knee flexion ROM 2 years after CR‐TKA.

## AUTHOR CONTRIBUTIONS

Conception and design of the study; acquisition, analysis and interpretation of data; drafting the article: Tatsuya Kubo. Conception and design of the study; acquisition, analysis and interpretation of data; critical revision of the article for substantial intellectual contribution: Tsuneari Takahashi. Critical revision of the article for substantial intellectual contribution: Katsushi Takeshita. All authors have contributed significantly to the study, approved the article, and consented to its submission. All authors reviewed and approved the final version of the manuscript.

## CONFLICT OF INTEREST STATEMENT

The authors declare no conflicts of interest.

## ETHICS STATEMENT

This research was conducted following the Declaration of Helsinki and was sanctioned by the Ishibashi General Hospital Bioethics Committee for Medical Research (Approval ID: 25‐NO.12). It involved a retrospective study. All patients underwent standard treatment, and obtaining informed consent from individual participants was not required.

## Data Availability

Data and materials for this study are available from the corresponding author upon reasonable request.
